# P80 natural essence spray and lozenges provide respiratory protection against Influenza A, B, and SARS-CoV-2

**DOI:** 10.1186/s12931-024-02718-0

**Published:** 2024-02-28

**Authors:** Viktoria Zaderer, Gabriel Diem, Wilfried Posch, Thomas Jakschitz, Günther K. Bonn, Rosa Bellmann-Weiler, Lukas A. Huber, Doris Wilflingseder

**Affiliations:** 1 ADSI - Austrian Drug Screening Institute GmbH, Innrain 66, Innsbruck, 6020 Austria; 2 Department of Internal Medicine II, Medical University of Innsbruck, Anichstraße 35, Innsbruck, Austria; 3 Institute of Cell Biology, Biocenter, Medical University of Innsbruck, Innrain 81/82, Innsbruck, 6020 Austria; 4 Institute of Hygiene and Medical Microbiology, Medical University of Innsbruck, Schöpfstraße 41, Innsbruck, 6020 Austria

**Keywords:** SARS-CoV-2, Influenza, Epithelial barrier model, Antiviral spray, Antiviral lozenges, P80

## Abstract

Seasonally circulating viruses, such as Influenza, as well as newly emerging viruses and variants thereof, and waning immunity urge the need for safe, easy-to-use and inexpensive drugs to protect from these challenges. To prevent transmission of these viruses and subsequent excessive inflammatory reactions on mucous membranes, we tested the efficacy of the natural essence P80 as spray and in form of lozenges against respiratory infections caused by SARS-CoV-2 variants of concern (VoCs), influenza A (H3N2) and influenza B (Victoria). P80 natural essence, a *Dimocarpus* longan extract, shielded highly differentiated human airway epithelia from SARS-CoV-2 wildtype and Omicron variant as well as Influenza A and B infection and dampened inflammation by down-modulating pro-inflammatory cytokine and anaphylatoxin secretion. A single application of P80 natural essence spray maintained tissue integrity long-term. This also significantly reduced the release of infectious viral particles and the secretion of IP10, MCP1, RANTES and C3a, all of which mediate the migration of immune cells to the sites of infection. Even P80 lozenges dissolved in distilled water or non-neutralizing saliva efficiently prevented SARS-CoV-2 and Influenza-induced tissue destruction. Consequently, our in vitro data suggest that P80 natural essence can act as antiviral prophylactic, both in form of nasal or oral spray and in form of lozenges, independent of circulating respiratory challenges.

**Supplementary Information:**

The online version contains supplementary material available at 10.1186/s12931-024-02718-0.

## Introduction

Emerging SARS-CoV-2 variants of concern (VoC) as well as Influenza A and B viruses are important respiratory pathogens occurring worldwide and causing – in particular in immune-compromised individuals – severe pathogenesis with high mortality rates (WHO). Vaccines against these respiratory challenges offer protection against clinical disease in healthy individuals (COVID-19 vaccines, Influenza vaccines), but quick, easy-to-use, and inexpensive measures are still needed to prevent viral transmission. Recently, we reported on the antiviral and dendritic cell (DC)-modulating capacities of the *Dimocarpus* longan extract P80 in relation to HIV-1 [1]. Thus, here, we tested the efficiency of P80 natural essence as spray and in form of lozenges against respiratory challenges such as SARS-CoV-2 variants of concern (VoCs), Influenza A (H3N2) and/or Influenza B (Victoria). The subtropical and tropical Longan plant (*Dimocarpus longan Lour.*) belongs to the *Sapindaceae* family. It has economic significance due to its use in the fruit industry as dried longan pulp, longan juice, longan jelly, longan wine, and canned longan in syrup, and is regarded as an immunomodulatory agent in medical approaches [1–6]. Natural medicinal plants have a number of advantages in the treatment of infectious diseases, such as their richness, little or no side effects, and bioavailability. Invading pathogens such as SARS-CoV-2, Influenza A and B first encounter the respiratory mucosal barrier, which they effectively infect via receptor-mediated interactions and consequently cause tissue destruction and a pronounced inflammatory response [7]. Rapid emergence of contagious novel SARS-CoV-2 VoCs, seasonal Influenza A and B outbreaks as well as waning immunity call for broadly available preventive measures and prophylactic treatments. Due to its already reported antiviral activity, P80 natural essence is particularly well suited as agent against various virus groups. The COVID-19 pandemic illustrated an urgent need for sophisticated, human tissue models to rapidly test and develop effective treatment options against respiratory challenges in general. Therefore, respiratory and pulmonary model development has seen a rapid increase over the last three years. Using these physiologically relevant models, SARS-CoV-2 and Influenza A or B infections can be studied as well as novel treatment options. We and others have demonstrated, after infection of highly differentiated, pseudostratified human airway epithelial (HAE) tissue cultures and lung organoids with SARS-CoV-2 VoCs, that substantial mobilization of intracellular complement-C3 occurs within the nonimmune epithelial barrier [8–12]. This was connected to subsequent anaphylatoxin and pro-inflammatory cytokine release, high viral loads and massive tissue destruction. Virus-mediated events at barrier sites were effectively blocked by either basolateral treatment using C5a receptor antagonists, Cilgavimab/Tixagevimab or apically applied sprays, such as ColdZyme® and GlyPerA™ [9, 11–13]. Therefore, we here apically applied P80 natural essence in form of a spray or as a lozenges dissolved in distilled water or antibody-depleted saliva to HAE tissue models and tested their efficacy against the SARS-CoV-2 wild type isolate, the highly contagious Omicron variant BA.5 as well as Influenza A and B.

## Materials and methods

### Longan extract

Longan extract (P80 natural essence) is a herbal extract from PM 80 Co., Ltd., Bangkok, Thailand (herbal registration number i6200134/62).

### Cell culture of tissue models and P80 natural essence treatment

#### Human Airway Epithelia (HAE)

Normal human bronchial epithelial (NHBE, Lonza, cat# CC-2540 S, Germany) are available in our laboratary and routinely cultured in air liquid interface (ALI) as described [9, 10, 14].

Briefly, cells were cultured in a T75 flask for 2–4 days until they reached 80% confluency. The cells were detached using TrypLE (Thermo Fisher Scientific) and seeded onto GrowDexT (UPM)-coated 0.33 cm^2^ porous (0.4 μm) polyester membrane inserts with a density of 1 × 10^5^ cells per Transwell (Costar, Corning, New York, NY, USA). The cells were grown to near confluency in submerged culture for 2–3 days in specific epithelial cell growth medium according to the manufacturer´s instructions (PneumaCult™-Ex Plus Medium, Stemcell, cat# 05040, Germany). Cultures were maintained in a humidified atmosphere with 5% CO_2_ at 37 °C and then transferred to ALI culture in PneumaCult™-ALI medium (Stemcell, cat# 05001, Germany) for another 30 days until fully differentiated.

#### Spray application

Routinely, one hub of 1% P80 natural essence spray was apically applied 30 min prior infection using SARS-CoV-2 wild type or Omicron BA.5, Influenza A (H3N2) or B (Victoria). Cells were infected at MOI of 0.01. The apical application by spraying was carefully performed so as not to mechanically destroy the epithelial surface. Before using the apical 1% P80 solution to treat tissues, the integrity of the tissue was with of 0.1% and 1% solution applied apically or basolaterally, as shown in Suppl. Fig. 1. Since the TEER values did not differ between 0.1% and 1% final P80 concentration, experiments were performed at 1% P80.

#### Lozenges application

For the experiments using the P80 lozenges, these were diluted at a final concentration of 1% in distilled water (P80 1% Ad) or saliva tested to not neutralizing SARS-CoV-2 or Influenza in plaque assays (P80 1% saliva). 25 µl of the diluted lozenges were applied to the HAE tissue models 30 min prior infection using SARS-CoV-2 wild type or Omicron BA.5 and Influenza B (Victoria). Cells were infected at MOI of 0.01. Care was taken not to mechanically injure the epithelial surface during apical pipetting.

### Viruses

Clinical specimen for SARS-CoV-2 wild-type (WT) and Omicron BA.5 (B.1.1.529 BA.5) from COVID-19-positive swabs, sequenced by the Austrian Agency for Health and Food Safety, Vienna, Austria were propagated in Vero cells and subsequently used to infect cells. Influenza viruses were obtained from BEI resources (BEI Resources, Manassas, VA, USA; CFAR/NIBSC; Nr-52,281, Nr-52,282, NR-52,286 / Influenza virus A; HongKong/2671/2019 NIBSC code:19/292 / Influenza B /Victoria lineage NIBSC code: 20/242) and propagated in MDCK cells according to the protocol provided.

#### Vero cells

VeroE6/TMPRSS2/ACE2 is an engineered VeroE6 cell line expressing high levels of TMPRSS2 and ACE2 and highly susceptible to SARS-CoV-2 infection. This cell line was used to expand characterized WT and BA.5 viruses from patient isolates. The cell line was obtained via the CFAR (NIBSC) and is described by Matsuyama et al. [14].

### TEER measurement

TEER values were measured using EVOM volt-ohm-meter with STX-2 chopstick electrodes (World Precision Instruments, Stevenage, UK). Measurements on cells in ALI culture were made immediately before the change of medium. For measurements, 0.1 ml and 0.7 ml of medium were added to the apical and basolateral chambers, respectively. Cells were allowed to equilibrate before TEER was measured. TEER values reported were corrected for the resistance and surface area of the Transwell filters. To test whether HAE cells could cope with P80 treatment, TEER values of HAE cells treated with PBS were compared with those of HAE cells treated apically or basolaterally with 1% and 0.1% P80; these looked similar regardless of the treatment used (Suppl. Fig. 1a).

### Real-time RT-PCR for absolute quantification of SARS-CoV-2 and influenza A and B

SARS-CoV-2, Influenza A and B RNA were extracted using FavorPrep Viral RNA Mini Kit, according to manufacturer’s instructions (Favorgen Europe, cat# FAVRE 96,004, Austria). Sequences specific to 2 distinct regions of the Nucleocapsid (N) gene, N1 and N2, for SARS-CoV-2, or matrix genes for Influenza A or B and for the detection of a human housekeeping gene, Ribonuclease P, were used. Single target assays of all 3 targets were performed in combination with the Luna Universal Probe One-Step RT-qPCR Kit (New England Biolabs, cat# E3006, Germany). For absolute quantification using the standard curve method, SARS-CoV-2 RNA was obtained as a PCR standard control from the National Institute for Biological Standards and Control, UK. All runs were performed on a Bio-Rad CFX 96 instrument and analyzed by the Bio-Rad CFX Maestro 1.1 software (Bio-Rad, Germany). Samples for viral quantification were taken apically and basolaterally on several days post infection (dpi).

### Staining and high content screening (HCS)

To visualize SARS-CoV-2 infection 3D tissue models, cells were infected with clinical specimen of SARS-CoV-2 wild type or Omicron and analyzed for characteristic markers in infection experiments on day 3 to 4 post infection (3 or 4 dpi). After SARS-CoV-2 exposure, 3D cell cultures were fixed with 4% paraformaldehyde. Intracellular staining was performed using 1x Intracellular Staining Permeabilization Wash Buffer (10X; BioLegend, San Diego, CA, USA). Nuclei were detected using Hoechst 33,342 (Cell Signaling Technologies, cat# 4082, Netherlands), complement C3 using a C3-FITC (Agilent Technologies, cat# F020102-2, Austria) and cilia using an acetylated Tubulin-Alexa647 antibody (Abcam, cat# ab218591, UK). In some experiments, actin was stained using Phalloidin. Intracellular SARS-CoV-2 was detected using Alexa594-labeled SARS-CoV-2 antibodies against S1 and N (both Sino Biological, Beijing, China). The Alexa594-labeling kit was purchased from Abcam, Cambridge, UK. After staining, 3D cultures were mounted in Mowiol. To study these complex models using primary cells cultured in 3D and to generate detailed phenotypic fingerprints for deeper biological insights in a high throughput manner, the Operetta CLS System (PerkinElmer, Waltham, MA, USA) was used. For analyzing nuclei counts, spot analyses for SARS-CoV-2 particles and C3 area, Harmony™ Software was used and analyses performed in more than 1500 cells per condition.

### Plaque assay

VeroE6/ACE2/TMPRSS2 cells were inoculated with serial dilutions (1:10 dilutions) of 2 dpi subnatants from Omicron subvariant- or Wildtype (WT)-infected HAE cells for 1 h at 37 °C/5%CO_2_. Inoculate was replaced with culture medium containing 1.5% carboxymethylcellulose and incubated for 3 days at 37 °C/5% CO_2_ before plaque visualization and counting as described [9].

### Statistical analysis

Statistical analysis of differences in infection levels, TEER values, imaging approaches, or cytokine production was performed utilizing the GraphPad prism software and using OneWay ANOVA with Tukey´s posttest or unpaired Student´s t test upon comparison of two groups.

## Results

### Long-term rescue of tissue integrity upon SARS-CoV-2 infection by pre-treatment with P80 natural essence spray

**Fig. 1:**
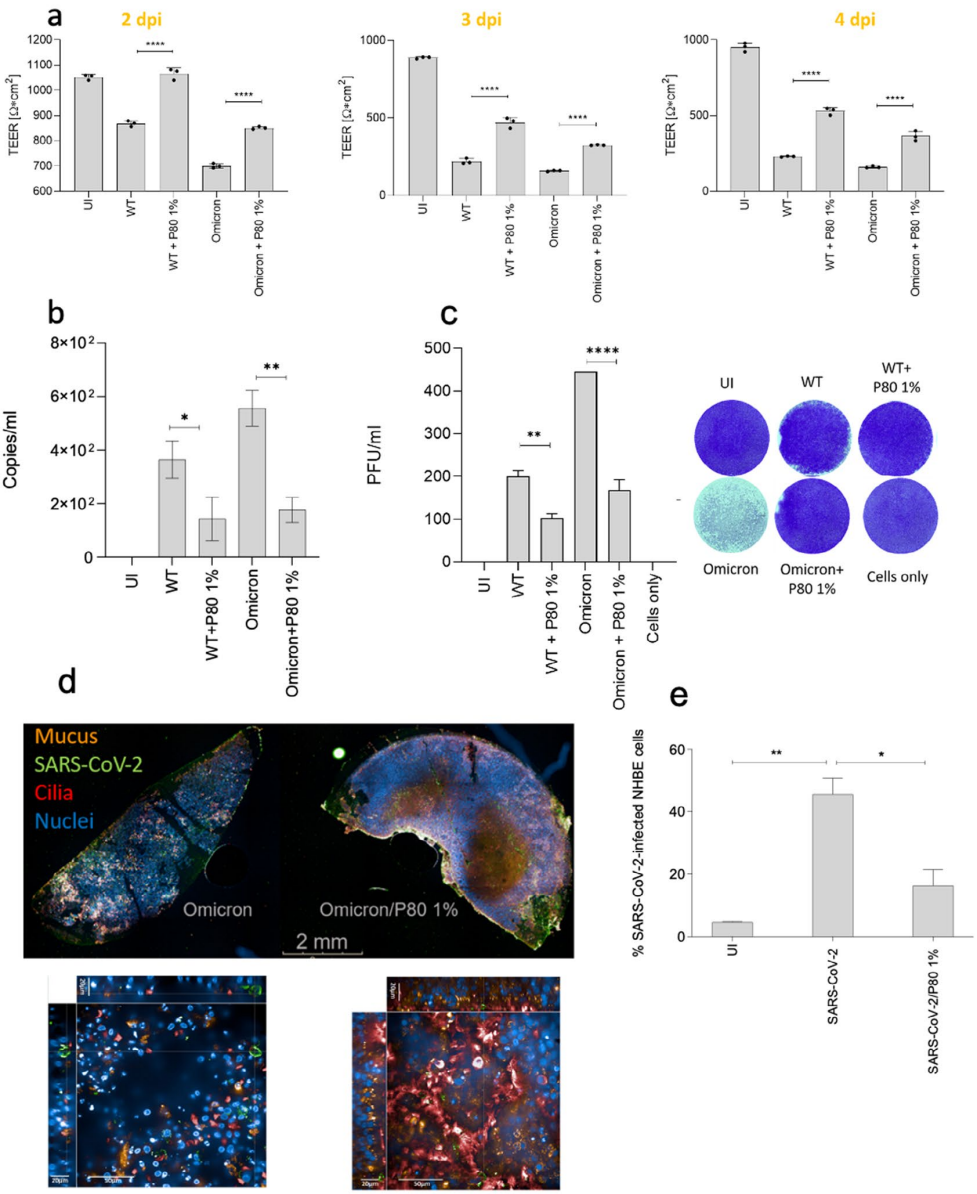
Disruption of epithelial integrity by SARS-CoV-2 WT and Omicron BA.5 can be avoided by pre-treatment with 1% P80 spray. (**a**) Pseudostratified epithelia were infected by apical addition of SARS-CoV-2 WT and BA.5 (MOI 0.01) with or without 1% P80 pretreatment and incubated for up to 96 h (4 dpi). Uninfected samples treated with 1% P80 were used as negative controls (UI). TEER was measured on 2 dpi (left), 3 dpi (middle) and 4 dpi (right) using an EVOM volt-ohm-meter. TEER in Ω/cm^2^ was determined for all conditions (UI, WT, WT + P80 1%, Omicron, Omicron + P80 1%) and plotted on a bar graph. Bars represent the mean + SD from 3 independent pseudostratified epithelia. Statistical significance was calculated using One-way ANOVA with Tukey´s multiple comparisons test. (**b**, **c**) Viral RNA **(b)** and plaques (**c**) were analyzed from subnatants of UI, WT, WT + P80 1%, Omicron, Omicron + P80 1% cultures of pseudostratified epithelia on 4 dpi. The experiment was repeated at least 3 times and statistically significant differences were determined by one-way ANOVA with Tukey´s multiple comparisons test. All values are means ± SD. Left, copy numbers are depicted, while the right panel shows plaque forming units (PFU) from 3 independent experiments (bar graph) and one representative plaque assay (images). (**d**) Immunofluorescence analyses of Omicron and Omicron/P80 1%-infected tissues are illustrated at a 5xAIR (upper panel) and 63xWater (lower panel) magnification. The 5x magnification shows an overview on the virus-infected tissue slides minus (left)/plus (right) 1% P80 pre-treatment, while the xyz view using the 63xW objective illustrates the tissue protection by P80 treatment (right) compared to solely infected (left) cultures. Tissues were stained using MUC5AC (Mucus, orange), SARS-CoV-2 N (SARS-CoV-2, green), acetylated tubulin (Cilia, red) and Hoechst (Nuclei, blue). (**e**) Percentages of SARS-CoV-2-infected HAE tissue samples using both, WT and Omicron variants (SARS-CoV-2), are depicted. At least 500 cells were quantified per condition, significant differences were determined using GraphPad Prism and one-way ANOVA

To test, whether a single administration of P80 natural essence, which can be applied as oral- or nasal spray, can provide long-term protection of airway tissues from SARS-CoV-2-mediated damage, HAE cultures were maintained in culture for four days after treatment and infection. Prior to performing tests using 1% P80, tests on epithelial integrity and mucociliary clearance were performed as described in the methods section and in Suppl. Fig. 1a and 1b. These tests revealed that 1% P80 had no negative effect on tissue integrity (Suppl. Fig. 1a) and significantly enhanced mucociliary clearance (Suppl. Fig. 1b and Suppl. Videos 1–4). The solution was added at a final concentration of 1% 30 min prior infection with SARS-CoV-2 wild type (WT) or Omicron BA.5 (Omicron) (MOI 0.01). Analyses of transepithelial electrical resistance (TEER; Fig. 1a) were performed up to 4 days post infection (4 dpi). These analyses revealed that pre-treatment with 1% P80 significantly rescued the tissue integrity in both, WT and Omicron-infected cultures, on 2, 3 and 4 dpi, compared to infected cells (Fig. 1a). Infection with WT illustrated TEER values comparable to P80-treated, uninfected controls (UI) up to 2 dpi, while values dropped on 3 dpi and 4 dpi compared to UI. In Omicron-infected cultures, a significant decrease compared to UI was also seen upon 1% P80 pre-treatment already on 2 dpi, and remained low on 3 dpi and 4 dpi (Fig. 1a). Still, all cultures pre-treated with 1% P80 showed a significantly higher TEER as solely infected cultures independent on the virus isolate used (Fig. 1a). Thus, in infection experiments, tissue integrity was significantly rescued up to 4 days post-infection when the natural essence spray P80 was administered only once prior to SARS-CoV-2 exposure.

### Significant decrease of SARS-CoV-2 viral loads and infectivity by pre-treatment with P80 natural essence spray

In accordance to TEER, absolute quantification of viral loads from 4 dpi subnatants of differently treated cells revealed protection from infection by administering the 1% P80 spray (Fig. 1b, left). Virus copy numbers in P80-treated and infected subnatants were slightly higher than background levels of mock-treated UI and significantly lower compared to WT- and Omicron-infected cultures (Fig. 1b). While in basolateral subnatants approximately 400 (WT) to 600 (Omicron) viral copies were detected, these values were 4 to 6 times lower in P80 natural essence-treated cultures (Fig. 1b). Moreover, infectivity of released virus particles from HAE cells were applied in a plaque assay and revealed similar results. While WT- and Omicron BA.5 subnatants depicted the highest infectivity corresponding to the viral load quantitation, pre-treatment of cultures with 1% P80 natural essence significantly reduced plaque formation (Fig. 1c). Figure 1c shows a summary of counted virus plaques from various donors on the left and plaques from one donor on the right. To refine analyses regarding tissue destruction and viral load, immunofluorescence data were generated using MUC5AC (mucus and mucus-producing cells, orange), acetylated tubulin (ciliated cells, red), Hoechst stain (nuclei, blue) and SARS-CoV-2 anti-N mAb (virus, green) (Fig. 1d) following infection with WT- (not shown) or Omicron BA.5 (Fig. 1d in absence or presence of 1% P80 natural essence. As revealed from TEER (Fig. 1a) and virus analyses (Fig. 1b and c), one hub of 1% P80 natural essence spray resulted in significant protection of the epithelial barrier (Figs. 1d and 5×, upper panel, and 63×W, lower panel, magnification of Omicron- and Omicron-P80-infected cultures; Suppl. Fig. #x00A0;2 3D analysis of infected versus P80-treated/infected cultures) as well as significantly lower percentages of SARS-CoV-2-infected cells (Fig. 1e; Suppl. Fig. 2 3D analysis of infected versus P80-treated/infected cultures). Similar results were obtained exposing the cells to SARS-CoV-2 WT or Omicron; thus, we combined data obtained from both, WT and Omicron BA.5 strains and the bar graph summarizes percentages of infected cells independent on the variant used (Fig. 1e, SARS-CoV-2, SARS-CoV-2/P80 1%). Here, we demonstrated that one hub of 1% P80 natural essence blocked SARS-CoV-2 infection and tissue destruction of HAE cultures independent on the SARS-CoV-2 variant used.

### Significant decrease of SARS-CoV-2-mediated induction of anaphylatoxin C3a and chemotactic proteins (IP10, MCP1, RANTES) by pre-treatment with P80 natural essence spray

**Fig. 2:**
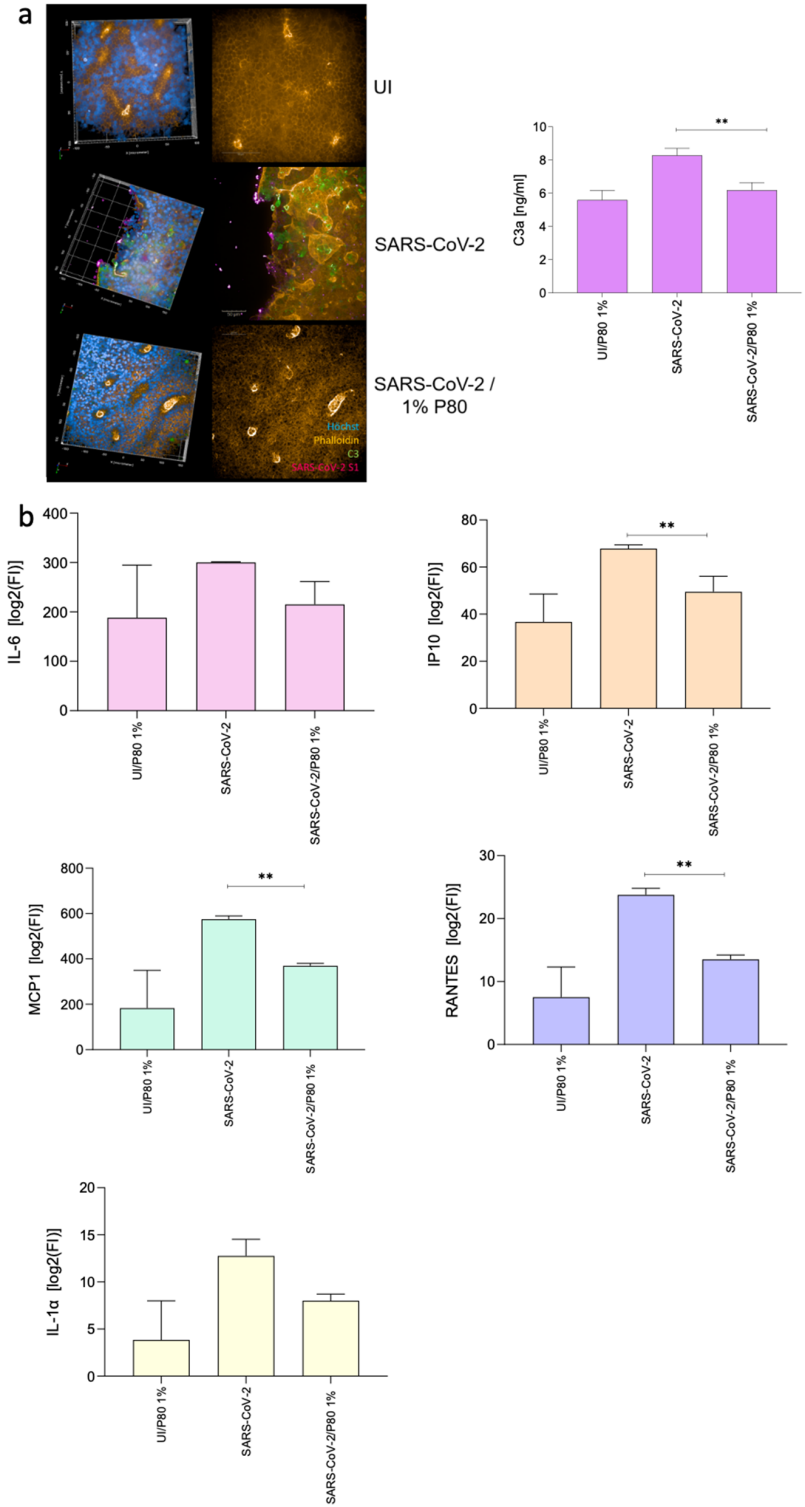
SARS-CoV-2 WT and BA.5 (SARS-CoV-2) significantly trigger innate immune activation from HAE cells that can be avoided by pre-treatment with 1% P80 spray. (**a**) *(Left)* Visualization of SARS-CoV-2 infection and C3 expression in 3D pseudostratified epithelia. Multilayered epithelia were infected using SARS-CoV-2. On 4 dpi, tissues were stained for Hoechst (blue), SARS-CoV-2 S1/N (pink), complement C3 (green) and phalloidin (orange). Representative 3D and input images are shown on the left from UI (and 1% P80 treated), SARS-CoV-2- and SARS-CoV-2/1% P80-treated samples. Here, tissues were infected using Omicron BA.5, but similar results were seen for WT infection. Experiments were performed at least 4 times and representative images are shown. *(Right)* C3a levels were determined for UI, SARS-CoV-2- and SARS-CoV-2/P80 1%-infected epithelial and plotted on a bar graph. Experiments were independently repeated 3 times using WT and Omicron variants and are summarized herein (SARS-CoV-2, SARS-CoV-2/ P80 1%). Data from WT and Omicron were combined and are presented independent on the variant used (SARS-CoV-2, SARS-CoV.2/P80 1%). (**b**) Supernatants from UI, SARS-CoV-2- or SARS-CoV-2/P80-infected pseudostratified epithelia were harvested 3 dpi and cytokine profiles determined using Luminex technology. Levels of cytokines are expressed in mean fluorescence intensities. Two independent experiments in duplicates were performed. Statistical significance was evaluated using One-way ANOVA and Dunnett´s post-test

Next, we measured anaphylatoxin and pro-inflammatory cytokine levels in mock-treated UI, infected and 1% P80 natural essence-treated and SARS-CoV-2 (WT and Omicron)-infected cultures (Fig. 2). As observed in Fig. 1c using a different staining (MUC5AC, acetylated tubulin, Hoechst stain, virus), tissue structures were maintained in P80-pre-treated and infected samples compared to solely infected ones as analyzed this time by phalloidin staining (orange) (Fig. 2a, left). Here, slides were stained using phalloidin (actin, orange), complement C3 (C3, green) for innate immune activation, Hoechst stain (nuclei, blue) and SARS-CoV-2 anti-N mAb (virus, pink) (Fig. 2a left, Suppl. Fig. 2). 3D analyses of UI, SARS-CoV-2- and P80/SARS-CoV-2 samples further revealed that beside significantly decreased virus signal (pink), local C3 (green) was produced to considerably lower levels in UI and P80/SARS-CoV-2 samples compared to infected ones (Fig. 2a, left). Figure 2a (left) shows a representative example of Omicron-infected cultures. Increased intracellular C3 mobilization in SARS-CoV-2-infected cultures was associated with enhanced basolateral C3a anaphylatoxin release, in both WT and Omicron-infected cultures (Fig. 2a, right, SARS-CoV-2). The release was significantly modulated down to levels of 1% P80-treated, uninfected control samples by treating the cultures with 1% P80 before SARS-CoV-2 infection (Fig. 2a, right). In addition to the decrease in C3a anaphylatoxin in samples treated with 1% P80 natural essence, there was a significant decrease in IP10, MCP1, and RANTES levels, all chemokines that mediate immune cell migration to sites of infection, compared to SARS-CoV-2 infected cultures (Fig. 2b, orange, green and lilac bar charts). Trends for P80-mediated drop in inflammatory processes during infection were further visible for IL-6 and IL-1α (Fig. 2b, pink and yellow bar charts), though these were not significant. In summary, we have shown here that pretreatment of differentiated HAE cultures with a 1% natural essence P80 spray can suppress SARS-CoV-2-mediated upregulation of local C3 mobilization as well as the release of anaphylatoxin C3a and the chemotactic proteins MCP-1, IP-10, and RANTES. Moreover, production of pro-inflammatory cytokines IL-6 and IL-1α were decreased in P80-treated and infected samples compared to infected ones.

### Long-term rescue of tissue integrity upon Influenza A (H3N2) and B (Victoria) infection by pre-treatment with P80 natural essence spray

**Fig. 3:**
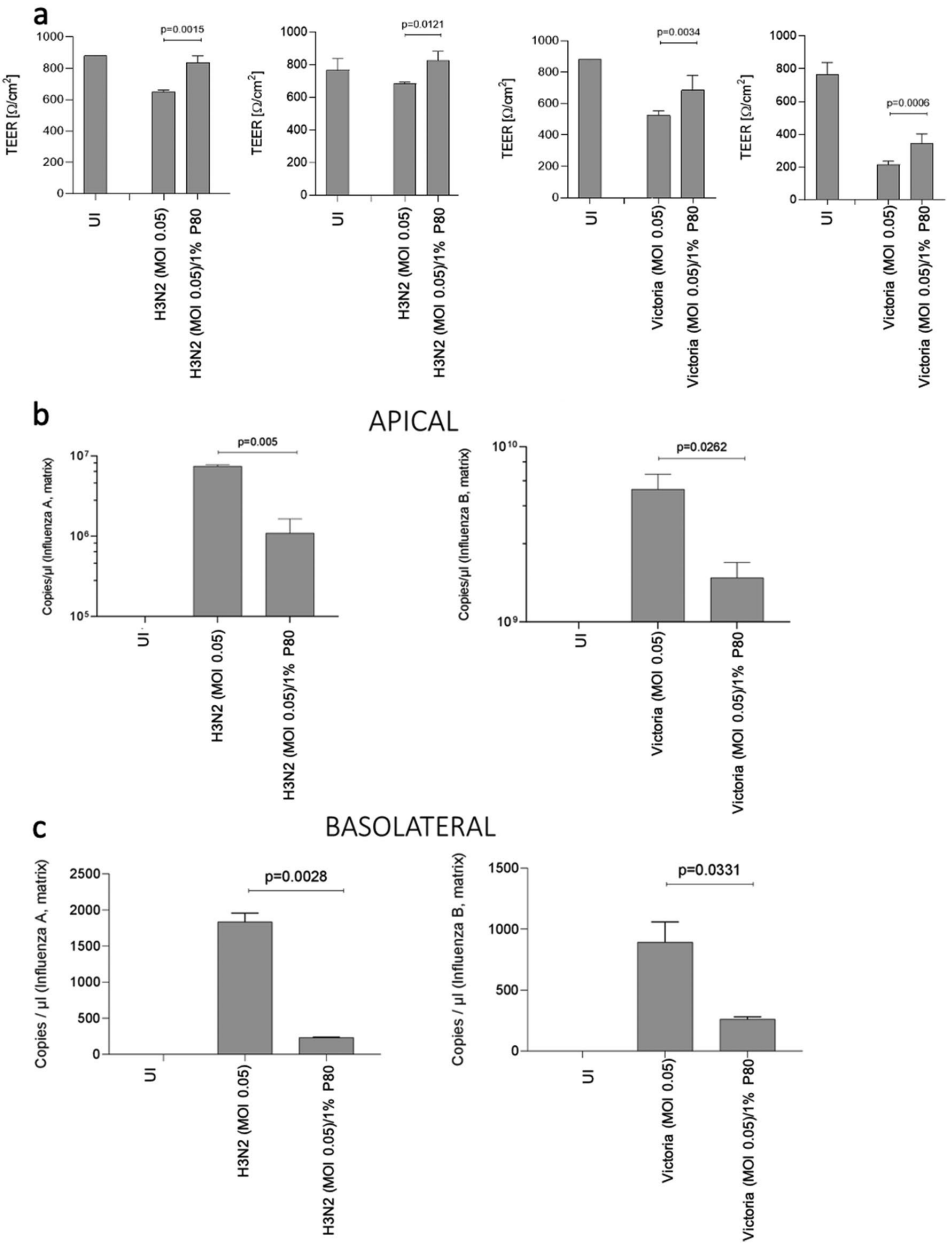
Pre-treatment with 1% P80 spray protects HAE from tissue disruption and infection by Influenza A and B. (**a**) Pseudostratified epithelia were infected by apical addition of Influenza A (H3N2, left) and B (Influenza B, right) at MOIs 0.05 with or without 1% P80 pretreatment and incubated for up to 72 h (3 dpi). Uninfected samples treated with 1% P80 were used as negative controls (UI). TEER was measured on 2 dpi *(left)*, 3 dpi *(middle)* and 4 dpi *(right)* using an EVOM volt-ohm-meter. TEER in Ω/cm^2^ was determined for all conditions (UI, H3N2, H3N2/1% P80, Influenza B, Influenza B/1% P80) 1 dpi (1^st^ and 3^rd^ panel) and 2 dpi (2^nd^ and 4^th^ panel) and plotted on a bar graph. Bars represent the mean + SD from 3 independent pseudostratified epithelia. Statistical significance was calculated using One-way ANOVA with Tukey´s multiple comparisons test. (**b**, **c**) Viral RNA from apical (**b**) and basolateral (**c**) were analyzed from subnatants of UI, H3N2, H3N2/1% P80, Influenza B, and Influenza B/1% P80 cultures of pseudostratified epithelia on 3 dpi. The experiment was repeated at least 3 times and statistically significant differences were determined by one-way ANOVA with Tukey´s multiple comparisons test. All values are means ± SD

Further, we analyzed, if the P80 natural essence spray is efficient to rescue from Influenza A and B infection. For this, we applied the spray at a final concentration of 1% 30 min prior infection with Influenza at a MOI of 0.05. On 2 and 3 dpi, TEER was measured as described before. These analyses revealed that pre-treatment with 1% P80 significantly rescued the tissue integrity in both, Influenza A (left, H3N2) and B (right)-infected cultures, on 2 and 3 dpi, compared to infected cells (Fig. 3a). Infection with Influenza A (H3N2) illustrated TEER values comparable to P80-treated, uninfected controls (UI) until 3 dpi, while for Influenza B values dropped already on 2 dpi and even more on 3 dpi compared to UI (Fig. 3a). Nevertheless, all cultures pre-treated with 1% P80 showed a significantly higher TEER as solely infected cultures independent on using Influenza A or B (Fig. 3a). Thus, tissue integrity was greatly rescued in infection experiments up to 3 days analyzed post infection, if P80 natural spray was administered only once prior Influenza A or B exposure.

### Significant decrease of SARS-CoV-2 viral loads and infectivity by pre-treatment with P80 natural essence spray

In accordance to TEER, absolute quantification of viral loads from apical and basolateral 3 dpi supernatants of differently treated cells revealed protection from Influenza A and B infection by administering the 1% P80 spray (Fig. 3b (apical virus release) and Fig. 3c(basolateral virus release)). Virus copy numbers in P80-treated and infected apical and basolateral supernatants were significantly lower in both, Influenza A (H3N2) and B (Influenza B) (Fig. 3b and c). Thus, P80 natural essence spray not only protected against SARS-CoV-2, but also significantly shielded airway epithelia from infection with Influenza A and B.

### Shielding of HAE from SARS-CoV-2 and Influenza B by P80 natural essence lozenges

**Fig. 4:**
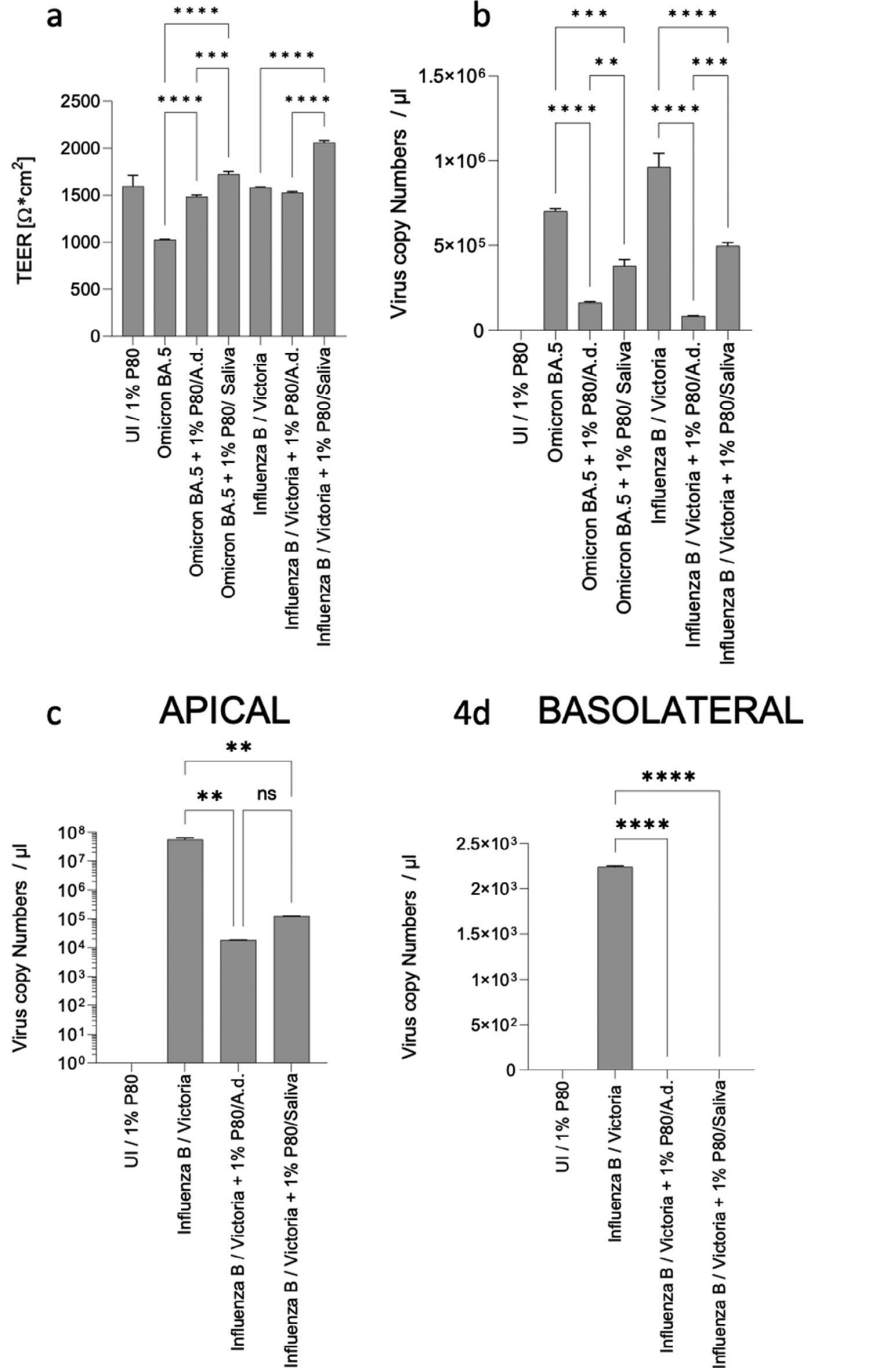
Pre-treatment with 1% P80 lozenges diluted in bidestilled water (A.d.) or non-neutralizing saliva protects HAE from tissue disruption and infection by Omicron BA.5 and Influenza B. (**a**, **b**) Pseudostratified epithelia pretreated or not with 1% P80 diluted in A.d. or saliva prior infection with SARS-CoV-2 Omicron BA.5 or Influenza B were analyzed for tissue destruction measuring TEER (**a**) and virus copy number by quantitative RT-PCR (**b**). Experiments were performed at least thrice in duplicates and statistical significance calculated using GraphPad Prism software and one-way ANOVA. (**c**, **d**) Viral RNA from apical (**c**) and basolateral (**d**) were analyzed from subnatants of UI, Influenza B, Influenza B + 1% P80/A.d. and Influenza B + 1% P80/Saliva cultures of pseudostratified epithelia on 3 dpi. The experiment was repeated at least 3 times and statistically significant differences were determined by one-way ANOVA with Tukey´s multiple comparisons test. All values are means ± SD

Finally, we tested the efficiency of P80 in form of lozenges instead of a spray, as this is an even easier and more convenient way to administer an antiviral agent. For this, the P80 lozenges were diluted to a concentration of 1% in either distilled water or non-neutralizing saliva to mimic the process in the body even more realistically. The diluted drops were applied to the HAE cells prior infection with SARS-CoV-2 or Influenza B at MOIs indicated above. One and two days post infection, TEER values were measured and viral load was determined in apical and basolateral supernatants. These analyses revealed that P80 natural essence lozenges significantly protected the airway epithelia from destruction (Fig. 4a) and also in terms of viral load (Fig. 4b) independent on the virus used (SARS-CoV-2, Influenza B) or the medium, in which the lozenges were diluted (A.d., saliva). PCR from apical and basolateral side was performed on 2 dpi for Influenza B and these analyses revealed that the P80 lozenges diluted in A.d. or saliva exerted a significant protection from apical (Fig. 4c,) and even more effective basolateral (Fig. 4d) virus release also 2 days post infection. Overall, these analyses showed that the natural essence P80 was antivirally effective even in lozenge form and in saliva.

## Discussion

The P80 natural essence from the subtropical and tropical Longan plant (*Dimocarpus longan* Lour.) illustrated potential immunomodulatory and antiviral mechanisms also in terms of Hepatitis C virus or HIV-1 [1–6]. Thus, here, the protective features of P80 natural essence were tested in form of a spray and as lozenges against the currently prevalent respiratory challenges, SARS-CoV-2 and Influenza. Earlier, ColdZyme®, low molecular weight heparin (LMWH) enoxaparin and GlyPerA™ solution, were shown to be potent in blocking SARS-CoV-2 variants of concern (VoCs) as mouth- and/or nasal spray or by inhalation (enoxaparin) [11–13, 15]. All tested solutions are ubiquitously available and easily applicable, which makes them good candidates for prophylactic treatment against SARS-CoV-2. In addition to the tested solutions, it would be desirable to find plant-derived medicinal products effective against respiratory challenges due to their multifaceted benefits, like their richness, low side effects and costs and high bioavailability. We found that P80 natural essence spray and lozenges were highly effective to prevent from SARS-CoV-2 wild type and Omicron BA.5 as well as Influenza A and B-mediated damage of human airway epithelial tissue models and rescued the respiratory tissues from productive infection with SARS-CoV-2 variants and Influenza A/B and from virus-mediated inflammatory processes. The NHBE model used is of primary origin, and is a very robust, predictive and standardized system optimal for testing first mechanisms of human respiratory viruses, like SARS-CoV-2 and Influenza, as well as the effectivity of antiviral compounds. We found that not only tissue integrity was rescued during SARS-CoV-2 and Influenza A/B infection by sole pre-treatment with 1% P80 natural essence, but this compound massively down-modulated C3 mobilization in HAE cells and associated release of the chemotactic anaphylatoxin C3a. Excessive complement activation has already been described by us and others in the first two waves of the SARS-CoV-2 pandemics in highly differentiated in vitro respiratory models, from bronchoalveolar lavages of patients and from reports of severe COVID-19 cases [8–13, 16, 17]. Targeting C3aR and C5aR at basolateral sides of highly differentiated pseudostratified human airway epithelia completely blocked the inflated local complement C3 activation and thus prevented intrinsic inflammation and tissue damage by down-modulating C3a levels, as well as proinflammatory cytokines IL-6, MCP1, IL-1α and RANTES [10]. We also found these effects on reducing chemotactic and inflammatory cytokines here by pretreating tissue models with SARS-CoV-2 wild-type or Omicron and with influenza A and/or B with 1% P80 spray or lozenges dissolved in saliva or distilled water before infection. The significant reduction of other chemotactic proteins, such as IP10, MCP1 and RANTES, which have been shown to be associated with the severity of COVID-19 infection in patients, will prevent the attraction of inflammatory immune cells to the sites of infection [18, 19]. This would be beneficial in preventing an exaggerated immune response leading to tissue destruction. The P80 natural essence will not only exert a positive effect on the non-immune epithelial barrier, but could further act as a dendritic cell (DC)-boosting adjuvant at barrier sites. In vitro analyses of DCs exposed to HIV-1 showed that the Longan natural essence had an antiviral and adjuvant effect on the sentinels of the human immune system and could therefore be applied as a mucosal adjuvant to provide a link between innate and adaptive immunity by exerting immune-stimulating effects on antigen-presenting cells [1]. Thus, these effects could prove beneficial also in respiratory infections. On the one hand P80 natural essence not only protected the epithelial barriers from respiratory infections, associated tissue destruction, virus production and overshooting inflammation, but could also activate virus-specific DCs at barrier sites that in turn prime/boost specific CD4^+^ and CD8^+^ T cell responses – this needs further studies within physiologically relevant immune-competent barrier models. The 1% concentration of P80 was chosen within this study due to its non-cytotoxic effects on DCs, which will next being added to the tissue environment to having even a more physiological, human primary model. This concentration of P80 natural essence was not only effective against SARS-CoV-2 VoC Omicron BA.5 and Influenza B as spray, but also when applied as saliva-dissolved lozenges – this would make the use even more flexible and comfortable compared to application via spraying or inhaling and can easily protect from virus transmission every time and everywhere. When applying lozenges dissolved in A.d. or saliva, we could show that both preparations protected from virus-mediated tissue destruction and significantly lowered viral loads within the tissue model, with lozenges dissolved in A.d. being even more effective in lowering copy numbers. This points to a small effect of proteins in saliva slightly weakening the P80-mediated effect, but still a highly significant reduction in apically and basolaterally released viral particles was observed. In summary, we found that both the spray and lozenges containing the natural essence P80 were highly effective in protecting against the transmission of SARS-CoV-2 wild-type and (spray)/or (lozenges) Omicron BA.5 and influenza A and (spray)/or (lozenges) influenza B in a highly relevant human primary model by restoring tissue integrity and blocking infection of highly differentiated HAE cells.

Furthermore, in SARS-CoV-2 wild-type and Omicron BA.5, we found that P80 natural essence protected against local complement activation and subsequent mobilization of chemotactic cytokines IP10, MCP1, and RANTES in particular, but also down-modulated proinflammatory cytokines IL-1α and IL-6. Although our data were obtained only in vitro and are therefore not directly transferable to efficacy in vivo, they open exciting new avenues that can be explored by using the natural essence P80 in lozenge form as an effective, safe, and widely available protection to limit the spread of respiratory viruses.

### Electronic supplementary material

Below is the link to the electronic supplementary material.

**Supplementary Material 1:** Testing effects of P80 treatment on HAE cultures

Supplementary Material 2

Supplementary Material 3

Supplementary Material 4

Supplementary Material 5

Supplementary Material 6

Supplementary Material 7

## Data Availability

All data generated or analyzed during this study are included in this article.
