# Supplementary material for: P80 natural essence spray and lozenges provide respiratory protection against Influenza A, B, and SARS-CoV-2
Source: Respir Res. 2024 Feb 28;25:102. doi: 10.1186/s12931-024-02718-0 (PMC10900741; doi:10.1186/s12931-024-02718-0)
Supplement: Supplementary file 1 — Supplementary Material 1: Testing effects of P80 treatment on HAE cultures [file 12931_2024_2718_MOESM1_ESM.pptx]

## Slide 1
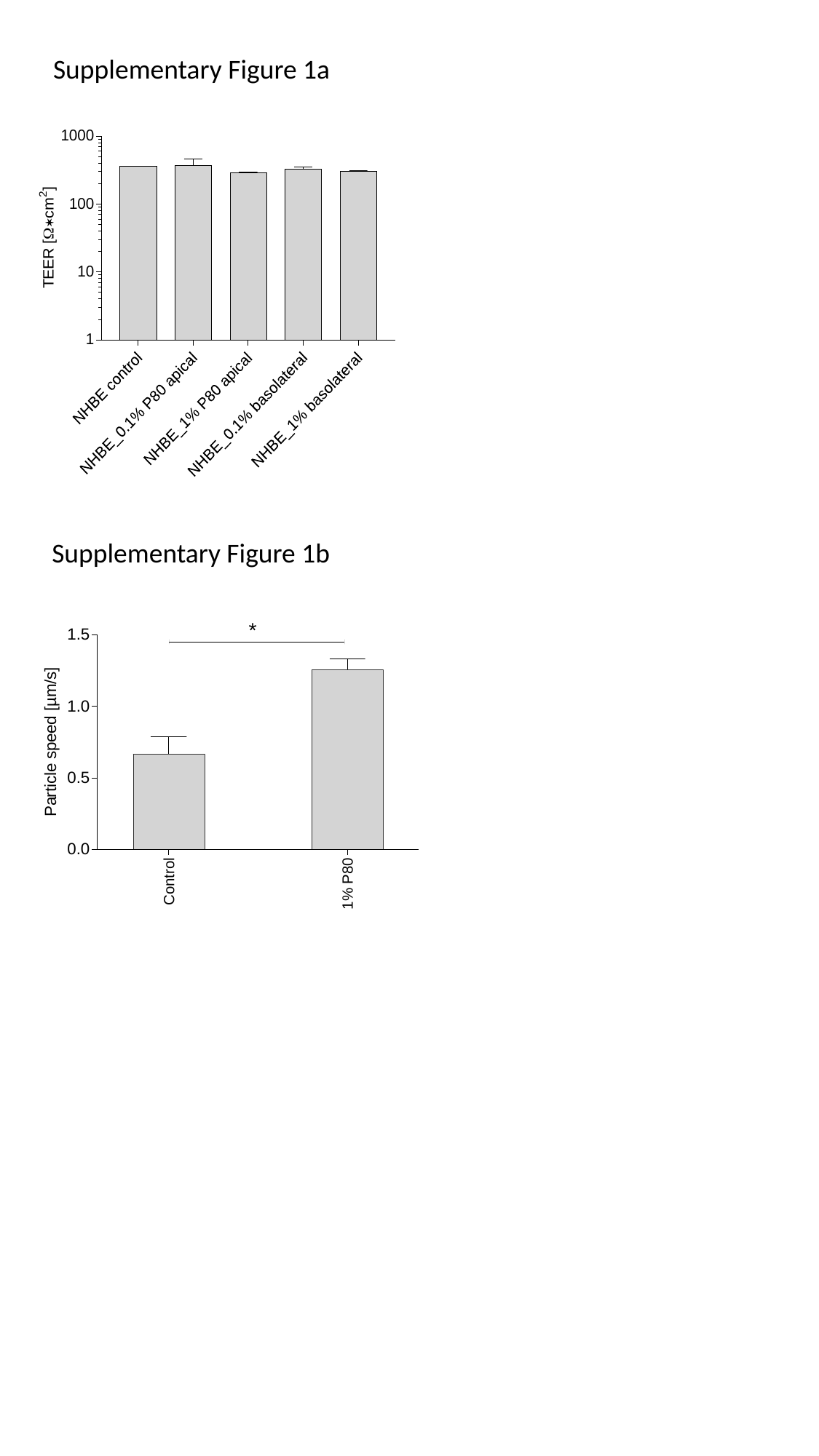

Supplementary Figure 1a
Supplementary Figure 1b

## Slide 2
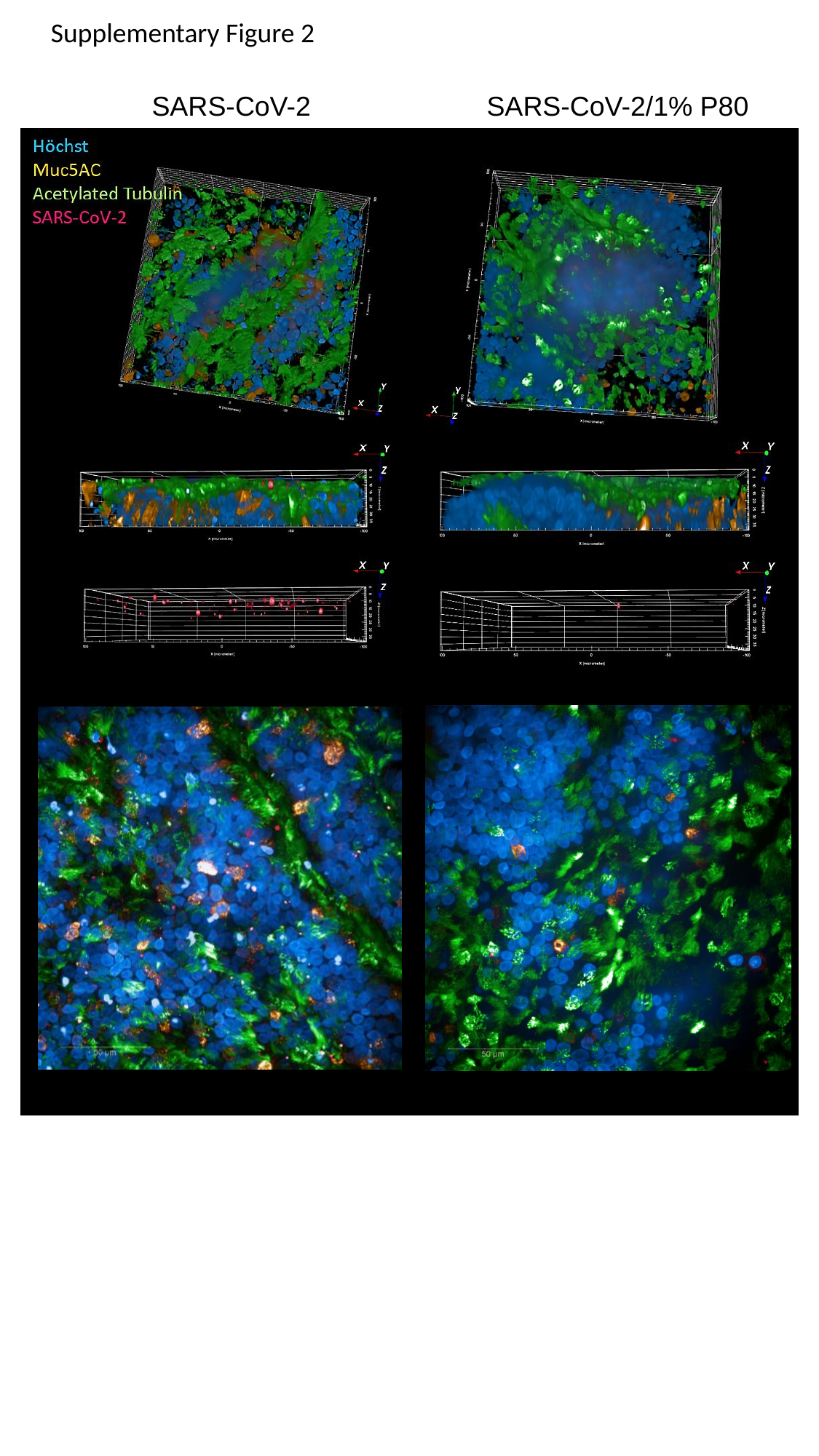

Supplementary Figure 2
SARS-CoV-2
SARS-CoV-2/1% P80
