# Supplementary material for: P80 natural essence spray and lozenges provide respiratory protection against Influenza A, B, and SARS-CoV-2
Source: Respir Res. 2024 Feb 28;25:102. doi: 10.1186/s12931-024-02718-0 (PMC10900741; doi:10.1186/s12931-024-02718-0)
Supplement: Supplementary file 6 — Supplementary Material 6 [file 12931_2024_2718_MOESM6_ESM.docx]

**Supplementary Figures**

**Suppl. Fig 1. (a) Testing tissue integrity of HAE cultures upon apical and basolateral treatment with 0.1% and 1% P80.** Pseudostratified epithelia were apically and basolaterally treated with 0.1% and 1% P80 to test, if tissue samples are affected by the treatments. TEER was measured on up to 3 days post treatment and a summary of three independent experiments of day 3 post treatment is depicted. No effects on epithelial integrity was observed. **(b) Mucociliary clearance is significantly accelerated upon treatment of HAE with 1% P80.** Pseudostratified epithelia were apically treated with 1% P80 and mucociliary clearance assessed using fluorescently labelled beads and live cell analyses as illustrated in Suppl. Videos 1 to 4. Mucociliary clearance was analyzed by measuring the speed of particles and results from three independent experiments are illustrated. The experiment was repeated at least 3 times and statistically significant differences were determined by one-way ANOVA with Tukey´s multiple comparisons test. All values are means ± SD.

**Suppl. Fig 2. 1% P80 rescues from infection and tissue destruction by SARS-CoV-2 (WT)**

Human airway epithelia (HAE) cells were infected with SARS-CoV-2 (WT) at an MOI of 0.01. 3 dpi cells were fixed and stained for Hoechst (blue), SARS-CoV-2 S1/N (red), acetylated tubulin (green) and mucus producing cells (orange) and then analyzed by confocal microscopy. The upper panel shows 3D images of SARS-CoV-2- (left) or SARS-CoV-2/1% P80 (right)-infected cells from apical (1^st^ panel) and lateral (2^nd^ panel) showing all stainings, as well as from lateral (3^rd^ panel) depicting solely the virus signal. While in WT-infected cultures the virus penetrated deep (3^rd^ panel, left), P80 pre-treatment protected from infection nearly completely and virus was only observed superficially (3^rd^ panel, right). This is also reflected in the input image of SARS-CoV-2 WT-infected cells (lower panel) that show virus signal (red) and small holes and more mucus-producing cells in the tissue and P80-pretreated and infected cells, which show an intact tissue, nearly no virus signal and low levels of mucus-producing cells. Experiments were repeated at least four times independently with similar results.

**Supplementary Videos**

**Suppl. Video 1. Analyses of D-PBS-treated HAE control cultures.** Cultures were treated with D-PBS spray and applied to live cell imaging using fluorescently labeled beads (orange), brightfield (BF) and Hoechst (blue) to assess mucociliary clearance (bead movement) and cells (BF and nuclei, blue). Live cell analyses were performed on an Operetta CLS system (Perkin Elmer) using the Harmony™ software.

**Suppl. Video 2. Tracking of fluorescently labeled beads in D-PBS-treated HAE control cultures.** Tracking of fluorescently labeled beads was performed on an Operetta CLS system (Perkin Elmer) using the Harmony™ software.

**Suppl. Video 3. Analyses of 1% P80-treated HAE cultures.** Cultures were treated with 1% P80 spray and applied to live cell imaging using fluorescently labeled beads (orange), brightfield (BF) and Hoechst (blue) to assess mucociliary clearance (bead movement) and cells (BF and nuclei, blue). Live cell analyses were performed on an Operetta CLS system (Perkin Elmer) using the Harmony™ software.

**Suppl. Video 4. Tracking of fluorescently labeled beads in 1% P80-treated HAE cultures.** Tracking of fluorescently labeled beads was performed on an Operetta CLS system (Perkin Elmer) using the Harmony™ software.
